# Effects of life-story review on quality of life, depression, and life satisfaction in older adults in Oman: a randomized controlled study

**DOI:** 10.1186/s12877-024-05133-8

**Published:** 2024-06-19

**Authors:** Bushra Rashid Al-Ghafri, Hamed Al-Sinawi, Ahmed Mohammed Al-Harrasi, Yaqoub Al-Saidi, Abdulaziz Al-Mahrezi, Zahir Badar Al-Ghusaini, Khalfan Bakhit Rashid Al-Zeedy, Moon Fai Chan

**Affiliations:** 1 Department of Family Medicine and Public Health, College of Medicine and Health Sciences, Sultan Qaboos University, Muscat, Oman; 2 Department of Behavioral Medicine, Sultan Qaboos University Hospital, Muscat, Oman; 3 Director General, Sultan Qaboos University Hospital, Muscat, Oman; 4 Department of Arabic and Literature, College of Arts, Sultan Qaboos University, Muscat, Oman; 5 Department of Internal Medicine, Sultan Qaboos University Hospital, Muscat, Oman

**Keywords:** Life-story review, Older adults, Life satisfaction, Quality of life, Depression

## Abstract

**Background:**

There is a need for healthcare providers to develop life-story review interventions to enhance the mental well-being and quality of life of older adults. The primary aim of this study is to examine the effects of telling their life stories and creating a life-story book intervention on QoL, depressive symptoms, and life satisfaction in a group of older adults in Oman.

**Methods:**

A repeated-measures randomized controlled design was conducted in Oman. A total of 75 older adults (response rate = 40.1%) were randomly assigned to the intervention (*n* = 38) or control (*n* = 37) groups. Demographic data were collected as the baseline. Depression, life satisfaction, and quality of life scores were collected from each participant at weeks 1, 2, 3, 4, and 8.

**Results:**

Their average age is 67.3 ± 5.5 years (range 60–82 years). There are more women (*n* = 50, 66.7%) than men. Over the 8 weeks, the intervention group exhibited a notable decrease in depression (intervention: 2.5 ± 1.2 vs. control: 5.3 ± 2.1, *p* < .001) but an increase in life satisfaction (24.6 ± 3.1 vs. 21.9 ± 6.1, *p* < .001) and quality of life (physical: 76.2 ± 12.7 vs. 53.6 ± 15.5, *p* < .001; psychological: 76.4 ± 12.1 vs. 59.9 ± 21.5, *p* < .001; Social relation: 78.3 ± 11.7 vs. 61.8 ± 16.6, *p* < .001; environment: 70.8 ± 10.2 vs. 58.6 ± 16.1, *p* < .001) compared to the control group.

**Conclusion:**

The life-story review intervention proved effective in diminishing depression and boosting life satisfaction and quality of life among the older sample within the 8-week study. Healthcare providers can apply such interventions to improve older adults’ mental health and well-being.

## Background

The World Health Organization has determined 4 domains for QoL: physical health, psychological, social relationships, and environment [1]. QoL in older adults is a critical aspect of senior care and is influenced by these domains. Each of these domains plays a crucial role in the overall QoL of older adults. It expands the definition of health to include a personal sense of physical and mental health, social functioning, and emotional well-being. Physical activity, living environment, and diet are key self-care behaviors contributing to health and QoL [2]. Jelicic & Kempen [3] examined 5,279 community-dwelling older adults and found that the lower the life quality is, the more chronic conditions older adults have. Physical health includes mobility, daily activities, functional capacity, energy, pain, and sleep [4]. Psychological well-being includes self-image, negative thoughts, positive attitudes, self-esteem, and mental status [5]. As depression levels rise, QoL levels in older adults will reduce [6], and when their life satisfaction levels rise, their QoL levels will improve [7]. A study in China found that higher life satisfaction of older adults can enhance the effects of social support on their social relationships, which reduces depressive symptoms [8]. A systematic review revealed 286 empirical studies showing that social networks and a sense of well-being are positively associated with life satisfaction in older adults [9]. QoL measures permit researchers to compare the status of different groups over time and assess the effectiveness of public health interventions [7]. Environmental factors also play a significant role in the QoL of older adults [5]. These four main factors influence the QoL of older adults, and understanding these can help develop strategies, care, and support to improve their QoL.

Previous researchers [10–13] have identified various factors influencing older adults’ life satisfaction. It has been found that participation in exercise or physical activities among older adults is a complementary factor that can boost life satisfaction levels [10]. A study in Poland found that seniors who participated were satisfied with their lives [11]. Another study in Oman, a 4-week follow-up study, surveyed a group of older adults in a community setting, and it reported no significant change in their life satisfaction levels [12]. However, a study reports that when depression levels rose, life satisfaction correspondingly decreased [11]. Another study of older adults in Korea reports that the living environment positively impacts life satisfaction [13]. These insights can be useful in enhancing life satisfaction levels by implementing suitable programs that promote healthy lifestyles among older adults [10].

Depression is a mental health condition that can affect how individuals feel, act, and think [14]. Depressive symptoms are a common problem among older adults, and they often go along with chronic illnesses that have significant impacts on their QoL [6, 15]. Previous studies have shown that many older adults reported lower QoL and life satisfaction but higher depressive symptoms [16]. A study in China of older adults found that depression reduces an older person’s physical activities and social network because they may lose interest in the things they normally enjoy [17]. A study in Oman reported a 16.9% prevalence rate of depression among Omani older adults, which is expected to be much higher in the upcoming decade due to an aging problem [18]. A qualitative study found that Omani older adults who are depressed or experiencing a crisis may suffer from emotional and bodily disturbances and find it difficult to express themselves verbally [19].

There is a need for healthcare providers to develop interventions to improve the physical health, mental well-being, and overall QoL of older adults. Sharing one’s life story has been recommended to alleviate depressive symptoms and enhance QoL and life satisfaction among older adults [20, 21]. A life-story work is a “*term given to biographical approaches in health and social care settings that give people time to share their memories and talk about their life experiences*” [22]. Wills & Day [23] asserted that people long to narrate their life stories, whether past or present, which correspond with their life experiences. These stories are influenced by social, political, and economic factors and culture, religion, and relationships, which confers a unique personality and identity [24, 25]. Some researchers suggested that reminiscence might provide a mechanism to facilitate adaptation and produce continuity in inner psychological characteristics, social behavior, and social circumstances [26]. Reminiscence is a psychosocial intervention focusing on remembering and sharing past stories that reinforce a sense of identity and self-worth. While reminiscence focuses on sharing stories and improving social interaction, life review aims to create a meaningful life story that integrates positive and negative experiences. A meta-analysis examining the effects of reminiscence interventions reveals a broad range of outcomes. Specifically, it shows moderate improvements in ego integrity and depression. However, the effects on purpose in life satisfaction and positive well-being were smaller than those in the control group [9]. Reminiscence and life review could empower individuals to find solace, meaning, and coherence while reflecting on their life journey. The foundational theoretical model for life review integrates the psychosocial development theory [27] and Butler’s life review theory [28]. This combined approach has been used in previous studies [16, 20] and forms the basis for the current study. By examining various life events, individuals can discover a sense of unity, purpose, and significance in their lives, reinforcing their identity and self-promotion [29]. The study findings revealed that older adults in the life story review group exhibited fewer depressive symptoms, reduced feelings of hopelessness, and improved life satisfaction compared to those in the control group [29]. This concept is encapsulated in the Theory of Narrative Identity [24]. Life-story books, typically filled with photos, keepsakes, and personal histories, are therapeutic by facilitating better communication among healthcare providers, clients, and relatives [30]. The study findings indicated that older adults who created life-story books experienced an increase in ego integrity, and their mental health showed signs of improvement after participating in the intervention [30]. Creating a life storybook offers a safe way to articulate deep-seated emotions and achieve emotional release [21]. Furthermore, no local research has been conducted on using life story reviews and creating life-story books as a healthcare intervention among older adults in Oman.

### Aim

The primary aim of this study is to examine the effects of telling their life stories and creating a life-story book intervention on QoL, depressive symptoms, and life satisfaction in a group of older adults in Oman.

### Hypotheses

1. There is a statistically significant difference in each outcome measure between groups during the 8-week study;
2. There is a statistically significant difference in each outcome measure on the 5 time points for each group.

## Methods

### Design

**Fig. 1:**
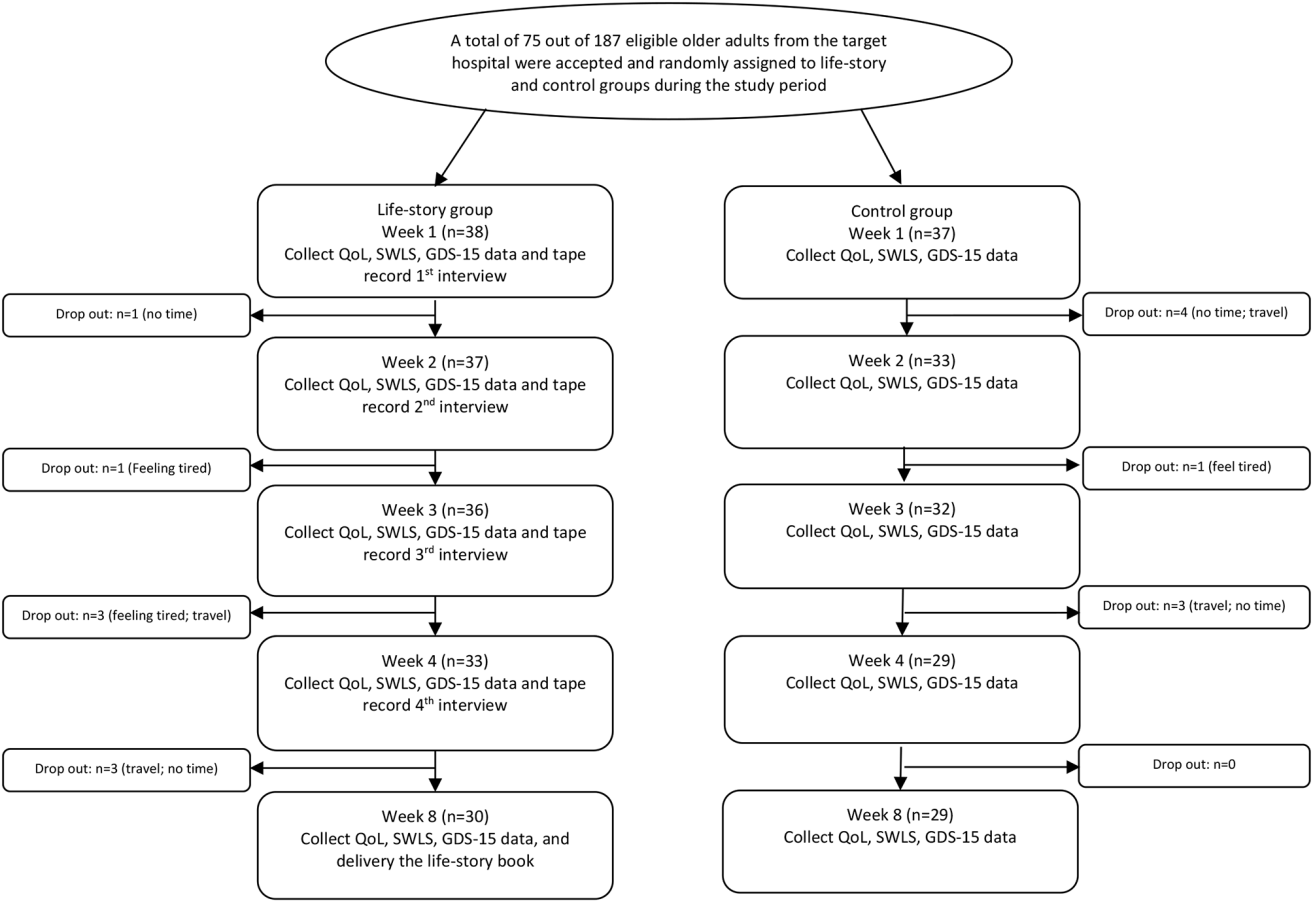
Flow chart of the study

This is a repeated-measures experimental randomized control design with two groups of older Oman adults living in the community. The flow chart of this study is shown in Fig. 1.

### Participants and ethical consideration

Inclusion criteria are Omani adults aged 60 or above who can communicate in Arabic or English and provide signed consent. In Oman, those aged 60 years and above were considered older adults [18, 19]. Those elderly who are diagnosed with Alzheimer’s disease, other neurocognitive disorders, Parkinson’s disease, or a major mental disorder, those using sleep medications, and those unwilling to be audio-recorded in the intervention group were excluded. This study was approved by the Sultan Qaboos University Medical Research Ethics Committee (MREC #2028), and all participants are required to provide written or verbal informed consent.

### Power analysis

The power analysis for this study is grounded on depression scores, one of the primary outcomes. A design involving repeated measures with two groups was employed, and the PASS statistical software was utilized to determine the necessary sample sizes [31]. Based on a prior meta-analysis conducted by our research team [32], this study anticipates effect sizes of 0.32, 0.47, and 0.47 for the between-group (2 groups), within-time (5 times) and interaction of the between-group and within-time factors, respectively. The study requires 38 subjects in each group, totaling 76 subjects. This will result in power levels of 79%, 92%, and 92% for the between-group, within-time, and interaction factors, respectively, at a 5% significance level.

### Outcome measures

The study instrument was divided into two parts:

#### Part 1

Demographic characteristics: Age, gender, marital status, educational level, and medical history were collected as baseline information for all participants [21, 22].

#### Part 2

Quality of life (QoL): The participants’ QoL levels were evaluated using the Arabic version of the World Health Organization quality of life questionnaire (WHOQoL-BREF) [33, 34]. It is a 26-item, and each item has a 5-point scale that is divided into 4 aspects (social relationships, physical, environment, and psychological) and two items on perceived “Satisfaction with health” and “General rating of QoL”. The Arabic version has an acceptable Cronbach’s alpha = 0.7 [34], and percentage scores range from 0 to 100, with higher scores indicating better quality of life in each domain and item.

Life satisfaction: The Arabic version of the Satisfaction with Life Scale (SWLS) [35] collected the participants’ life satisfaction levels. This tool has good reliability (*a* = 0.86) and is composed of 5 questions, each with a response option on a scale from 1 (strongly disagree) to 7 (strongly agree). The overall score can vary between 5 and 35, with a higher score indicating higher life satisfaction.

Depressive symptoms: The participants’ depression levels were measured by the Arabic version of the Geriatric Depression Scale (GDS-15) [36]. This tool has good reliability (a = 0.88) and includes 15 fixed-response questions that gauge the emotional state of older adults over the past week. The cumulative score is calculated, with higher totals reflecting more intense symptoms of depression.

### Data collection procedure

After ethical approval from the University, the recruitment will take place at the inpatient clinics of the Sultan Qaboos University Hospital. A researcher, the first author, was present in the clinics and extended invitations to potential subjects while they awaited their appointments. Subjects willing to join the study were randomly assigned to either an intervention or a control group. The first author was trained by an experienced healthcare professional, the co-author. She also asked to conduct a pilot study by recruiting 1 subject per group to ensure the data collection and logistic process follows the protocol. The researcher explained the roles to each participant in the study, and written consent was obtained before the interviews. Participants’ identities were protected because all data were identified only by case numbers. Participants were told that they could withdraw from the study at any time. For both groups, outcomes were collected at five time points: baseline (week 1), week 2, week 3, week 4, and week 8.

### Intervention group

The intervention group had five meetings, as detailed in Fig. 1. The first four meetings were home interviews, where the older participants reviewed one life stage each: childhood, adolescence, adulthood, and current life. The guiding questions for each stage were based on Chan et al. [20], Erikson’s [27], and Butler’s [28] theories of psychosocial development. The researchers drafted the story from the verbatim transcripts after each meeting. During the interviews, participants are prompted with guiding questions to encourage them to express their emotions and share their narratives. After the interviews were completed, transcripts were created and consolidated. The participants proofread the story in the next meeting, which enhanced their memory and agency [23]. At the fourth meeting, the participants saw and revised their life-story book with their chosen photos. At the fifth meeting, the participants received their life storybooks.

### Control group

The control and intervention groups had the same encounter times. However, the life-story interview was exclusive to the intervention group; the control group did not participate in this activity. Participants were required to complete assessments for three outcome measures in these meetings. To avoid studying contamination, the researcher minimized other conversations or discussions with subjects about life issues.

### Randomization

Participants who fulfilled the eligibility criteria and expressed interest in participating were chosen from the hospital’s outpatient clinics. Each participant was given a unique identification number. Based on factors such as age and gender, they were randomly allocated to either the intervention or control group. An online software, Research Randomizer [37], generated a list of 38 unique numbers ranging from 1 to 76. Participants whose identification numbers matched those on the list were assigned to the intervention group, while the remaining participants were placed in the control group. This is not a double-blind or single-blind study because the participants and the researcher know who receives a life story or control group.

### Statistical analysis

The analysis was divided into three parts. Part one uses descriptive statistics (e.g., mean, percentage, standard deviation) to explore the profile of the participants. Part two employs univariate analysis (e.g., t-test, c² test) to compare demographic characteristics between the two groups to ensure the homogeneity of their demographic characteristics. Part three is to address the two main hypotheses of this study. Considering that the outcomes we collected were time-dependent and demographic characteristics could potentially influence these outcomes, we employed a Generalized Estimating Equation (GEE) model. The GEE is a well-known method for analyzing longitudinal data, and it does not necessitate complete data at all time points. No imputation is required to replace missing values for further analysis [38]. In addition, the GEE model permitted the inclusion of demographic factors (e.g., gender, age) that could be used to adjust the results of each outcome [21]. The Wald χ^2^ statistics were used to examine any significant differences between groups at each time point and within times for each group for each outcome. All analyses were conducted using IBM SPSS v23, and a 5% significance level was set for this study.

## Results

### Participant characteristics

Out of 187 eligible older adults, 75 participated in the study, resulting in a response rate of 40.1% between October 2021 and November 2023. The primary reasons for refusal included lack of interest, time constraints, and family members’ restrictions. Thirty-eight and thirty-seven were randomly allocated to the intervention and control groups. However, 16 (8 from the intervention group and 8 from the control group) dropped out due to personal reasons, including no time or going for long travel. Table 1 shows the median age of participants is 67.0, and the range is 60–82 years, with an average age of 67.3 ± 5.5 years. There are more women (*n* = 50, 66.7%) than men. More than 78% (*n* = 59) of them had to take medication due to chronic diseases. For those with chronic illness, 71.2% (*n* = 42) had diabetes mellitus, 69.5% (*n* = 41) suffered from hypertension, 52.5% (*n* = 31) had cholesterol, and 18.6% (*n* = 11) suffered from cardiovascular diseases. Participants met their relatives (*n* = 54, 72.0%) at least once a week, but 53.4% (*n* = 40) met friends less than once a week. In addition, more than half of them (*n* = 30) do not do any physical activity every week. The demographic characteristics of the two groups exhibited no statistically significant differences (Table 1).

**Table 1:** Comparison of socio-demographic characteristics of the study sample by groups

Demographic	Total (*n* = 75) *n* (%)	Control (*n* = 37) *n* (%)	Life-story (*n* = 38) *n* (%)	Test statistics (*p*-value)
Gender				
Male	25 (33.3)	12 (32.4)	13 (34.2)	0.027^a^ (0.870)
Female	50 (66.7)	25 (67.6)	25 (65.8)	
Marital status				
Married	48 (64.0)	25 (67.6)	23 (60.5)	0.403^a^ (0.525)
Divorced/Widow	27 (36.0)	12 (32.4)	15 (39.5)	
Age (years)				
60–64	27 (36.0)	16 (43.2)	11 (28.9)	1.794^a^ (0.408)
65–69	22 (29.3)	9 (24.3)	13 (34.2)	
70+	26 (34.7)	12 (32.4)	14 (36.8)	
Mean ± SD	67.3 ± 5.5	66.7 ± 5.8	68.0 ± 5.1	1.022^b^ (0.310)
Median [Range]	67.0 [60–82]	65.0 [60–82]	69.0 [60–79]	
Educational level				
Illiterate	25 (33.3)	14 (37.8)	11 (28.9)	0.678^a^ (0.712)
Grade 1–6	33 (44.0)	15 (40.5)	18 (47.4)	
Grade 7+	17 (22.7)	8 (21.6)	9 (23.7)	
Occupation				
Retired	28 (37.3)	11 (29.7)	17 (44.7)	2.030^a^ (0.362)
Housekeeping	33 (44.0)	19 (51.4)	14 (36.8)	
Others (e.g., volunteer work)	14 (18.7)	7 (18.9)	7 (18.5)	
Living with family members				
No	9 (12.0)	7 (18.9)	2 (5.3)	3.266c (0.086)
Yes	66 (88.0)	30 (81.1)	36 (94.7)	
Take medication				
No	16 (21.3)	9 (24.3)	7 (18.4)	0.389^a^ (0.533)
Yes	59 (78.7)	28 (75.7)	31 (81.6)	
Chronic disease				
No	16 (21.3)	9 (24.3)	7 (18.4)	0.389^a^ (0.533)
Yes	59 (78.7)	28 (75.7)	31 (81.6)	
Diabetes Mellitus (Yes)	42 (71.2)	21 (75.0)	21 (67.7)	
Hypertension (Yes)	41 (69.5)	21 (75.0)	20 (64.5)	
Cholesterol (Yes)	31 (52.5)	14 (50.0)	17 (54.8)	
Cardiovascular (Yes)	11 (18.6)	5 (17.9)	6 (19.4)	
Meet with relatives				
Less than once a week	21 (28.0)	13 (35.2)	8 (21.0)	4.061^a^ (0.131)
Once a week	33 (44.0)	12 (32.4)	21 (55.3)	
More than once a week	21 (28.0)	12 (32.4)	9 (23.7)	
Meet with friends				
Less than once a week	40 (53.4)	21 (56.8)	19 (50.0)	0.891^a^ (0.641)
Once a week	13 (17.3)	7 (18.9)	6 (15.8)	
More than once a week	22 (29.3)	9 (24.3)	13 (34.2)	
Physical activity per week				
No	40 (53.3)	17 (45.9)	23 (60.5)	1.601^a^ (0.206)
Yes	35 (46.7)	20 (54.1)	15 (39.5)	

a, χ^2^ test; b, independent t-test; c, Fisher’s Exact test; Life-story: Intervention; SD, standard deviation

### The effect of the life-story Intervention on depression and life satisfaction compared with the control over time

**Fig. 2:**
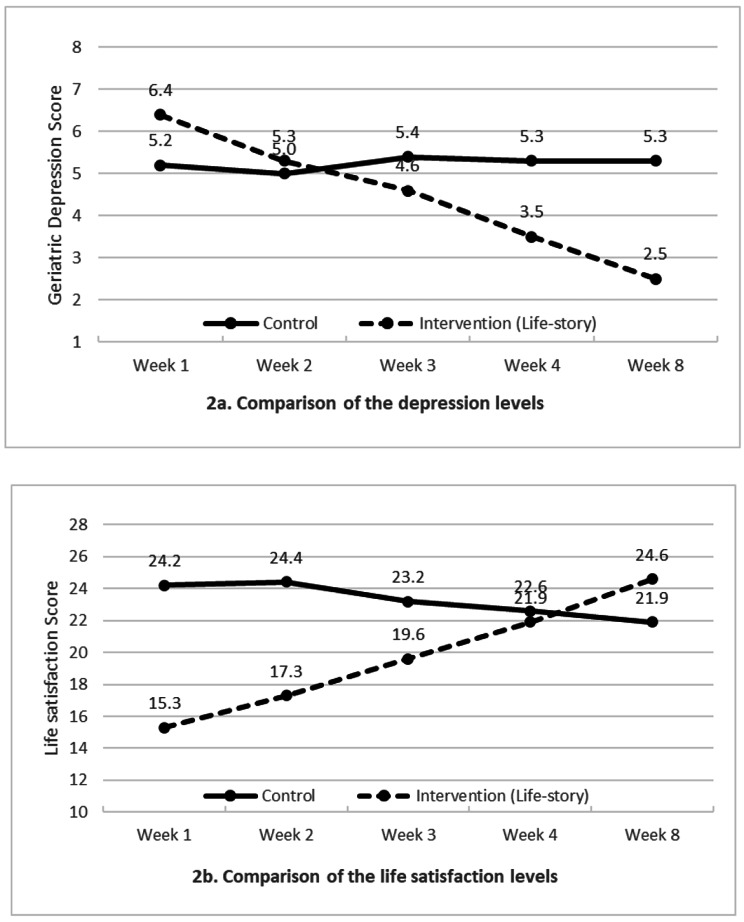
Comparison of the average depression and life satisfaction levels between older adults in the life-story and control groups

In Table 2, the baseline measures of depression levels between the two groups were compared. The intervention group (6.4 ± 0.7) had a significantly higher depression level than the control group (5.2 ± 1.7, t = 4.107, *p* < .001). Still, the GEE results show that there is a greater reduction in depression levels in older adults in the intervention group than in the control group on week 4 (Intervention: 3.5 ± 0.9 vs. Control: 5.3 ± 1.7, χ^2^ = 83.583, *p* < .001) and week 8 (2.5 ± 1.2 vs. 5.3 ± 2.1, χ^2^ = 108.726, *p* < .001). Within weeks, the depression score in the life-story group reduced dramatically (baseline vs. week 4, *p* < .001; baseline vs. week 8, *p* < .001), while the depression score in the control group remained with no significant change (baseline vs. week 4, *p* = .550; baseline vs. week 8, *p* = .850) (Fig. 2a).

On the life satisfaction scores, in the beginning, participants in the intervention group had a significantly lower life satisfaction level (15.3 ± 3.1) than the control group (24.2 ± 4.5, t = 9.967, *p* < .001). In the GEE analysis, the life-story group obtained a significantly higher score than the control groups in week 4 (Intervention: 21.9 ± 2.8 vs. Control: 22.6 ± 5.5, χ^2^ = 101.870, *p* < .001) and week 8 (24.6 ± 3.1 vs. 21.9 ± 6.1, χ^2^ = 167.846, *p* < .001). Over 8-week period, the life satisfaction score in the life-story group improved from week 1 (15.3 ± 3.1) to week 4 (21.9 ± 2.8, *p* < .001) and week 8 (24.6 ± 3.1, *p* < .001), while the life satisfaction score in the control group remained unchanged statistically from baseline (24.2 ± 4.5) to week 4 (22.6 ± 5.5, *p* = .253) until in week 8 (21.9 ± 6.1, *p* = .023). However, these changes were lower than the intervention group (Fig. 2b).

**Table 2:** GEE analysis to compare the depression and life satisfaction levels between groups and within times

Outcome	Control Mean ± SD	Life-story Mean ± SD	Test statistics^ (*p*-value)
**GDS-15**			
Week 1 (Baseline)	5.2 ± 1.7	6.4 ± 0.7	4.107^a^ (< 0.001**)
Week 2	5.0 ± 2.0	5.3 ± 0.9	4.940^b^ (0.026*)
Week 3	5.4 ± 1.8	4.6 ± 1.0	42.627^b^ (< 0.001**)
Week 4	5.3 ± 1.7	3.5 ± 0.9	83.583^b^ (< 0.001**)
Week 8	5.3 ± 2.1	2.5 ± 1.2	108.726^b^ (< 0.001**)
Test#, *p*-value	0.254^c^, 0.637^d^, 0.550^e^, 0.850^f^	< 0.001**^c, d, e, f^	
**SWLS**			
Week 1 (Baseline)	24.2 ± 4.5	15.3 ± 3.1	9.967^a^ (< 0.001**)
Week 2	24.4 ± 4.7	17.3 ± 3.2	9.739^b^ (0.002*)
Week 3	23.2 ± 5.1	19.6 ± 3.2	41.235^b^ (< 0.001**)
Week 4	22.6 ± 5.5	21.9 ± 2.8	101.870^b^ (< 0.001**)
Week 8	21.9 ± 6.1	24.6 ± 3.1	167.846^b^ (< 0.001**)
Test#, *p*-value	0.132^c^, 0.460^d^, 0.253^e^, 0.023*^f^	< .001^c, d, e, f^	

GEE, Generalized estimating equation, adjusted by age, gender, education level, physical activity, living with family members, medicine, and marital status; ^, GEE test between groups (Control vs. Life-story) at each time point; #, GEE test within 5 time points for each group; SD, standard deviation; GDS-15, Geriatric Depression Scale, 15 items, ranging from 0 to 15, higher the score showing depression; SWLS, Satisfaction with Life Scale, 5 items, ranging from 5 to 35, higher the score, showing greater life satisfaction; Week 1 (Control: *n* = 37, Intervention: *n* = 38); Week 2 (Control: *n* = 33, Intervention: *n* = 37); Week 3 (Control: *n* = 32, Intervention: *n* = 36); Week 4 (Control: *n* = 29, Intervention: *n* = 33); Week 8 (Control: *n* = 29, Intervention: *n* = 30); a, Independent t-test; b, Wald χ^2^; c, week 1 vs. week 2; d, week 1 vs. week 3; e, week1 vs. week 4; f, week 1 vs. week 8; *, Sig., at *p* < .05; **, Sig., at *p* < .001

### The effect of the life-story intervention on quality of life compared with the control over time

Quality of life (WHOQOL-BREF) scores were compared between the intervention and control groups (Table 3). There are 4 sub-domains (physical, psychological, social relationship, and environment). The general quality of life and satisfaction with health score were used to compare the two groups over an 8-week study.

**Table 3:** GEE analysis to compare the quality of life and sub-domain level between groups and within times

WHOQOL-BREF		Control Mean ± SD	Life-story (*n*) Mean ± SD	Test statistics^ *p*-value
***Physical domain***				
Week 1 (Baseline)		55.9 ± 15.9	46.9 ± 11.7	2.745^a^ (0.008*)
Week 2		55.6 ± 16.5	52.4 ± 12.1	9.262^b^ (0.002*)
Week 3		57.9 ± 15.7	62.0 ± 13.2	32.682^b^ (< 0.001**)
Week 4		54.9 ± 15.2	71.4 ± 10.7	104.589^b^ (< 0.001**)
Week 8		53.6 ± 15.5	76.2 ± 12.7	127.000^b^ (< 0.001**)
**Test#**, ***p*-value**		0.888^c^, 0.158^d^, 0.891^e^, 0.494^f^	< 0.001**^c, d, e, f^	
***Psychological domain***				
Week 1 (Baseline)		65.2 ± 17.3	44.8 ± 8.6	6.421^a^ (< 0.001**)
Week 2		61.4 ± 16.5	51.8 ± 7.5	14.268^b^ (< 0.001**)
Week 3		62.1 ± 15.4	59.6 ± 9.7	42.467^b^ (< 0.001**)
Week 4		62.2 ± 18.2	67.9 ± 10.2	68.629^b^ (< 0.001**)
Week 8		59.9 ± 21.5	76.4 ± 12.1	114.337^b^ (< 0.001**)
**Test#**, ***p*-value**		0.373^c^, 0.362^d^, 0.702^e^, 0.188^f^	< 0.001**^c, d, e, f^	
***Social relationship domain***				
Week 1 (Baseline)		66.7 ± 16.2	63.6 ± 8.1	1.034^a^ (0.306)
Week 2		66.9 ± 14.1	65.1 ± 9.4	0.351^b^ (0.554)
Week 3		64.8 ± 14.6	69.9 ± 9.8	9.356^b^ (0.002*)
Week 4		64.4 ± 13.3	74.0 ± 11.0	21.945^b^ (< 0.001**)
Week 8		61.8 ± 16.6	78.3 ± 11.7	33.745^b^ (< 0.001**)
**Test#,** *p* **-value**		0.898^c^, 0.560^d^, 0.522^e^, 0.133^f^	< 0.001**^c, d, e, f^	
***Environment domain***				
Week 1 (Baseline)	60.7 ± 12.6		51.5 ± 9.5	3.587^a^ (0.001**)
Week 2	61.5 ± 15.2		55.2 ± 7.9	21.211^b^ (< 0.001**)
Week 3	59.7 ± 14.3		60.2 ± 8.5	2.333^b^ (0.127)
Week 4	61.0 ± 14.5		66.4 ± 8.5	40.086^b^ (< 0.001**)
Week 8	58.6 ± 16.1		70.8 ± 10.2	70.498^b^ (< 0.001**)
**Test#,** *p* **-value**	0.282^c^, 0.883^d^, 0.275^e^, 0.735^f^		< 0.001**^c, d, e, f^	
***The general quality of life***				
Week 1 (Baseline)	62.8 ± 21.7		53.3 ± 18.5	2.044^a^ (0.045*)
Week 2	71.2 ± 18.9		47.3 ± 7.9	4.685^b^ (0.030*)
Week 3	66.4 ± 18.6		64.6 ± 12.5	1.436^b^ (0.231)
Week 4	69.0 ± 18.5		72.0 ± 10.4	3.321^b^ (0.068)
Week 8	66.4 ± 21.4		80.8 ± 12.6	9.917^b^ (0.002*)
**Test#** ***p*** **-value**	0.173^c^, 0.585^d^, 0.276^e^, 0.615^f^		< .001^c, d, e, f^	
***Satisfaction with health***				
Week 1 (Baseline)	55.4 ± 22.9		50.7 ± 23.6	0.883^a^ (0.380)
Week 2	68.2 ± 21.9		50.7 ± 16.1	2.934^b^ (0.087)
Week 3	68.0 ± 19.3		62.5 ± 14.0	0.013^b^ (0.909)
Week 4	62.9 ± 15.8		68.2 ± 12.9	1.796^b^ (0.180)
Week 8	55.2 ± 22.5		75.0 ± 13.1	9.758^b^ (0.002*)
**Test#** ***p*** **-value**	0.027*^c^, 0.019^d^, 0.140^e^, 0.997^f^		0.960^c^, 0.009*^d^, < 0.001**^e, f^	
GEE, Generalized estimating equation, adjusted by age, gender, education level, physical activity, living with family members, medicine, and marital status; ^, GEE test between groups (Control vs. Life-story) at each time point; #, GEE test within 5 time points for each group; SD, standard deviation; WHOQOL-BREF, The World Health Organization quality of life questionnaire, 26 items, ranging from 0 to 100 with higher scores indicating better quality of life in each sub-scale/domain; Week 1 (Control: *n* = 37, Intervention: *n* = 38); Week 2 (Control: *n* = 33, Intervention: *n* = 37); Week 3 (Control: *n* = 32, Intervention: *n* = 36); Week 4 (Control: *n* = 29, Intervention: *n* = 33); Week 8 (Control: *n* = 29, Intervention: *n* = 30); a, Independent t-test; b, Wald χ^2^; c, week 1 vs. week 2; d, week 1 vs. week 3; e, week1 vs. week 4; f, week 1 vs. week 8; *, Sig., at *p* < .05; **, Sig., at *p* < .001.				

**Fig. 3:**
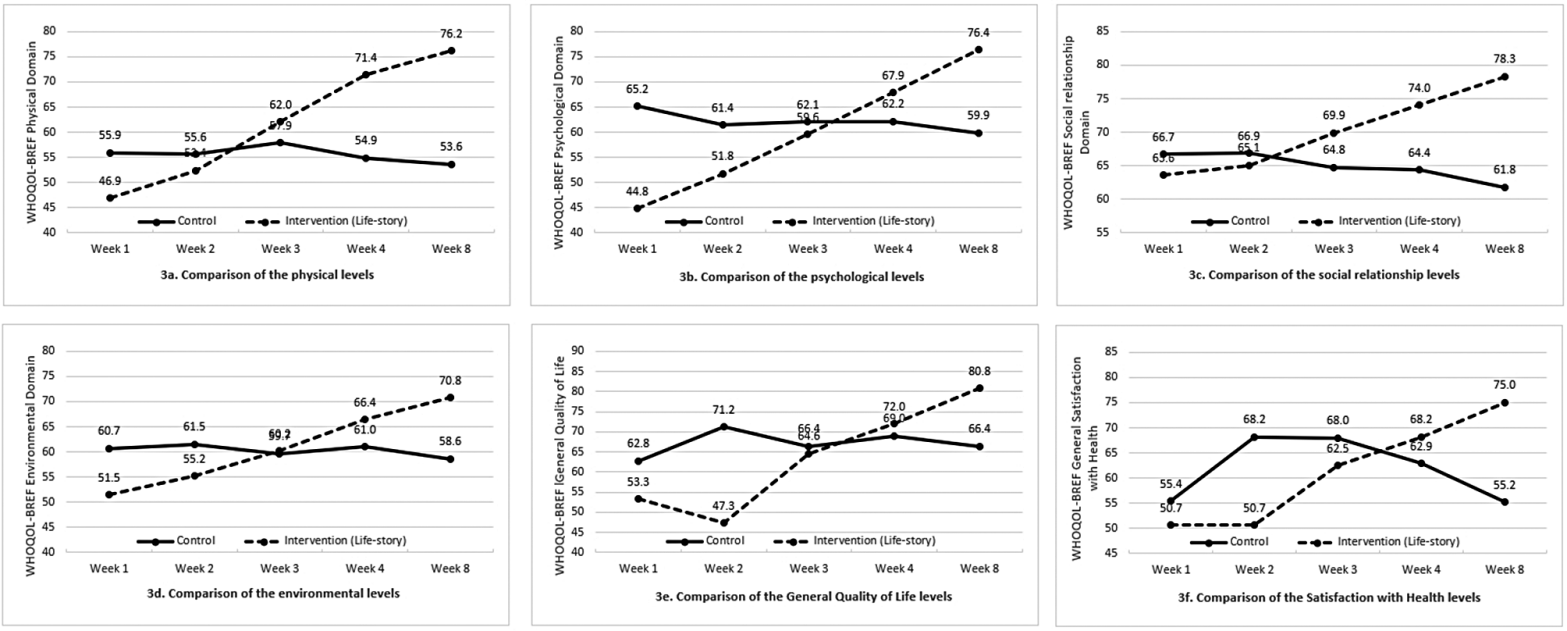
Comparison of the average quality of life (WHOQOL-BREF) between older adults in the life-story and control groups

In the physical domain, the life-story group obtained a significantly higher score than the control groups during the 8-week study, especially in week 4 (71.4 ± 10.7 vs. 54.9 ± 15.2, χ^2^ = 104.589, *p* < .001) and week 8 (76.2 ± 12.7 vs. 53.6 ± 15.5, χ^2^ = 127.000, *p* < .001). Within 8 weeks, older adults’ physical domain score in the life-story group improved dramatically from baseline (46.9 ± 11.7) to week 8 (76.2 ± 12.7, *p* < .001), while no significant change (baseline: 55.9 ± 15.9 vs. week 8: 53.6 ± 15.5, *p* = .494) on the control group (Fig. 3a).

In the psychological domain, a significantly higher score was found in the control (65.2 ± 17.3) than in the life-story group (44.8 ± 8.6, t = 6.421, *p* < .001) in the baseline. However, the psychological scores were significantly improved in the life-story group (week 4: 67.9 ± 10.2; week 8: 76.4 ± 12.1) than the control group (week 4: 62.2 ± 18.2, χ^2^ = 42.467, *p* < .001; week 8: 59.9 ± 21.5, χ^2^ = 114.337, *p* < .001) during the 8-week study. Over the 8 weeks, older adults’ psychological domain scores in the life-story group improved dramatically (baseline vs. week 8, *p* < .001). At the same time, there was no significant change (baseline vs. week 8, *p* = .188) in the control group (Fig. 3b).

In the social relationship domain, there was significantly more improvement in the intervention group (week 4: 74.0 ± 11.0; week 8: 78.3 ± 11.7) than the control group in week 4 (64.4 ± 13.3, χ^2^ = 21.945, *p* < .001) and week 8 (61.8 ± 16.6, χ^2^ = 33.745, *p* < .001) during the 8-week study. The social relationship scores in the life-story group improved from baseline (63.6 ± 8.1) to week 8 (78.3 ± 11.7, *p* < .001), while no significant change (baseline: 66.7 ± 15.2 vs. week 8: 61.8 ± 16.6, *p* = .133) on the control group (Fig. 3c).

In the environment domain, a significant improvement was found in the life-story group (week 4: 66.4 ± 8.5; week 8: 70.8 ± 10.2) compared to the control group in week 4 (61.0 ± 14.5, χ^2^ = 40.086, *p* < .001) and week 8 (58.6 ± 16.1, χ^2^ = 70.498, *p* < .001). Over the 8 weeks, the environment score in the life-story group improved from baseline (51.5 ± 9.5) to week 8 (70.8 ± 10.2, *p* < .001), while no significant change (baseline: 60.7 ± 12.6 vs. week 8: 58.6 ± 16.1, *p* = .735) in the control group (Fig. 3d).

In the general quality of life score, a significantly higher score was found in the control (62.8 ± 21.7) than in the life-story group (53.3 ± 18.5, t = 2.044, *p* = .045) in the baseline. However, a significant improvement was found in the older adults in the intervention group (80.8 ± 12.6) than in the control group (66.4 ± 21.4, χ^2^ = 9.917, *p* = .002) in week 8. Within time, older adults’ general quality of life score in the life-story group improved dramatically (baseline vs. week 8, *p* < .001). At the same time, there was no significant change (baseline vs. week 8, *p* = .615) in the control group (Fig. 3e).

In the satisfaction with health score, no significant difference was found between the control (55.4 ± 22.9) and the life-story group (50.7 ± 23.6, t = 0.883, *p* = .380) in the baseline. In the 8-week study, a significantly higher score was found for the older adults in the intervention group (75.0 ± 13.1) than the control group (55.2 ± 22.5, χ^2^ = 9.758, *p* = .002) in week 8. Over 8 weeks, the satisfaction with health score in the life-story group improved from baseline to week 8 (*p* < .001). In contrast, no significant reduction (baseline vs. week 8, *p* = .997) was found in the control group (Fig. 3f).

## Discussion

The conclusive report highlights that creating life-story books effectively mitigates depression and enhances life satisfaction and overall quality of life for older adults in the Omani community. Specifically, participants in the life-story group experienced significant improvements in life satisfaction compared to their counterparts in the control group after initiating the life-story book creation process. The use of narrative intervention, including reminiscence and life review, has demonstrated its efficacy as a powerful therapeutic approach for addressing depressive symptoms in older adults [39]. However, a prior study reported no significant effect of life review therapy on life satisfaction [40]. However, that study differed from ours in that it involved severely depressed older adults who were on psychiatric medication. Our research aligns with the studies conducted by Ligon et al. [41] and Chan et al. [20], which investigated verbal reminiscence therapy’s effects on older adults’ well-being using a design that included initial, subsequent, and follow-up evaluations. The findings revealed that the differences between the control and experimental groups became statistically significant after 10 weeks. In our study, the older adults were not on any psychiatric medication, and they engaged in a life-story book-making process that involved recalling both positive and negative memories. Erickson [27] proposed that looking back on one’s life is the last and final developmental task. Through this process of retrospection, the older adults in our study may have achieved a sense of fulfillment and wholeness [19]. Therefore, they may have developed a sense of purpose and happiness over time [13]. Our study posits that the narrative intervention was advantageous for the participants. Additionally, the life-story review was carried out through individual and one-to-one sessions at the participants’ residences, offering a secure space conducive to emotional expression and release.

Cully et al. [42] suggested that a life story review helps older adults process and release pent-up emotions, which can lessen depression. Our research indicates a notable decrease in depression among the older adults of the life-story intervention over 8 weeks, aligning with similar findings in the Chinese [16] and Malaysian [21] populations. Westerhof and Slatman [43], in their meta-analysis on life-story review, found that reducing depressive symptoms can consequently improve life satisfaction because this can improve someone’s mental well-being and can contribute to an increase in life satisfaction. Previous studies explain this kind of improvement because participants shared their past experiences and emotions with the researcher during the process, which can help alleviate feelings of sadness [15, 28]. This study found that those participants in the life-story group experienced appreciation and understanding when engaging with compassionate listeners. This interaction appears to substantially improve their life satisfaction and instill a deeper sense of value in their lives [44]. We understand that the baseline outcome on life satisfaction and depression levels are different between groups. In the study, the intervention group demonstrated better outcomes than the control group in terms of depression levels. The control group’s average depression scores remained consistent throughout the assessment sessions, while those of the intervention group decreased. If the baseline depression levels were similar between the two groups, the results would be more significant and robust. Similarly, for life satisfaction, the control group’s scores declined across sessions, whereas the intervention group’s scores reached the baseline levels of the control group. Again, if baseline differences were negligible, the results would be more significant and unaffected by these variations.

When conducting life story interventions in Oman, it is crucial to consider the religious and socio-cultural factors that may influence the study results. Oman is deeply rooted in Islamic traditions, which profoundly shape social norms, values, and behaviors. For instance, religious practices like praying 5 times daily can impact how people look at their life satisfaction and QoL [45]. Additionally, the concept of fate in Islam may affect older adults’ attitudes towards life satisfaction [46]. Oman typically has extended and closely-knit family structures, emphasizing collective well-being over individualism [47, 48]. Community cohesion and support networks are also vital aspects of Omani culture. Social gatherings, mutual aid, and community-based health initiatives are common, so they may not feel lonely and have fewer depressive symptoms [46]. Understanding these issues can help in designing the content of the life-story review interventions that ensure better acceptance and engagement [47, 49]. Including these religious and socio-cultural factors could help to improve the content of the life-story review intervention, which should be more likely to be more effective in Oman.

Conducting a life-story review and creating a life-story book can significantly improve the QoL in older adults. Our study results are consistent with previous research focusing on a life review or a life-story book-making process, improving older adults’ mental health and well-being [30]. This process may enhance the participant’s general well-being, which may help reduce their loneliness [20]. We found that making a life-story book influenced depression and life satisfaction, which enhanced the QoL of the intervention group participants, perhaps because of the cumulative benefits of this activity [16, 41]. Life-story work promotes attributing meaning to one’s life cycle, enhancing a sense of purpose and fulfillment [27]. It encourages social interaction and engagement, leading to a greater sense of belonging and improved social relationships [7]. It also improves psychosocial well-being, which can lead to higher QoL [17]. During the sessions, they may have resolved negative emotions related to different life stages, which may have led to gradual improvements in their mental health and well-being scores [24]. In conclusion, life-story work can positively impact older adults’ QoL by enhancing their cognitive and psychosocial well-being, increasing life satisfaction, and reducing depressive symptoms.

### Implications for clinical practice

The study emphasized early and effective intervention to enhance older adults’ well-being. This intervention could also prove valuable for older adults in community hospitals or day-care centers. By reviewing life and creating personal life-story books, healthcare professionals can gain deeper insights into each patient’s unique experiences, enabling tailored care to improve their QoL significantly [20]. It also adds to the current understanding of how producing life-story books as part of a life review can positively influence life satisfaction and depression among older adults. However, this result was based on a relatively small sample size study, so they should be interpreted cautiously. More studies need to focus on the progressive effects of similar interventions on older people.

### Limitations of this study

There are a few limitations that may affect the results of this study. First, these findings are based on a small sample size, and local studies are scarce to support them, indicating the need for further research. Second, this study had many female participants, which may not accurately represent the male population. It could be beneficial to conduct separate analyses for each gender on every outcome measure. However, more studies focusing on the male population are recommended due to the small sample size, particularly among male participants. Third, this study’s participants were recruited from the capital, which may not represent the entire Omani population. Some tribes still reside in the desert, and their traditional cultures and beliefs may differ from those living in the capital, suggesting the need for more studies in rural areas. Fourth, due to the inability to blind both participants and researchers, the Hawthorne effect could potentially influence the study results. Fifth, the current intervention’s benefits lasted 8 weeks; future studies should investigate its long-term effects on older adults over extended periods.

## Conclusions

This study showed statistically significant reductions in depression and improved life satisfaction and quality of life in older Omani adults in the life-story group compared to the control group. Conducting a life-story review and creating a life-story book is an effective intervention to alleviate the quality of life of older adults. Primary healthcare providers can guide older adults in adopting this method as a form of self-care, facilitating emotional release and fostering a therapeutic process in their everyday lives.

## Data Availability

The dataset used in this research can be made available with a reasonable request from the corresponding author.

## Abbreviations

QoL: Quality of life (WHOWOL-BREF)
GDS-15: Geriatric Depression Scale – Short form 15 items
SWLS: Satisfaction with life scale
GEE: Generalized Estimating Equation
